# Route to high-$T_{c}$ superconductivity of $\hbox {BC}_{{7}}$ via strong bonding of boron–carbon compound at high pressure

**DOI:** 10.1038/s41598-020-75049-x

**Published:** 2020-10-22

**Authors:** Prutthipong Tsuppayakorn-aek, Xiaoyong Yang, Prayoonsak Pluengphon, Wei Luo, Rajeev Ahuja, Thiti Bovornratanaraks

**Affiliations:** 1 Extreme Conditions Physics Research Laboratory (ECPRL) and Physics of Energy Materials Research Unit, Department of Physics, Faculty of Science, Chulalongkorn University, Bangkok, 10330 Thailand; 2 Thailand Centre of Excellence in Physics, Ministry of Higher Education, Science, Research and Innovation, 328 Si Ayutthaya Road, Bangkok, 10400 Thailand; 3 Condensed Matter Theory Group, Department of Physics and Materials Science, Uppsala University, Box 530, 751 21 Uppsala, Sweden; 4 Division of Physical Science, Faculty of Science and Technology, Huachiew Chalermprakiet University, Samutprakarn, 10540 Thailand; 5 Applied Materials Physics, Department of Materials and Engineering, Royal Institute of Technology (KTH), 100 44 Stockholm, Sweden

**Keywords:** Chemistry, Materials science, Physics

## Abstract

We have analyzed the compositions of boron–carbon system, in which the $\hbox {BC}_{{7}}$ compound is identified as structural stability at high pressure. The first-principles calculation is used to identify the phase diagram, electronic structure, and superconductivity of $\hbox {BC}_{{7}}$. Our results have demonstrated that the $\hbox {BC}_{{7}}$ is thermodynamically stable in the diamond-like $P{\bar{4}}m2$ structure at a pressure above 244 GPa, and under temperature also. Feature of chemical bonds between B and C atoms is presented using the electron localization function. The strong chemical bonds in diamond-like $P{\bar{4}}m2$ structure are covalent bonds, and it exhibits the s–p hybridization under the pressure compression. The Fermi surface shape displays the large sheet, indicating that the diamond-like $P{\bar{4}}m2$ phase can achieve a high superconducting transition temperature ($\hbox {T}_{{c}}$). The outstanding property of $\hbox {BC}_{{7}}$ at 250 GPa has manifested very high-$\hbox {T}_{{c}}$ of superconductivity as 164 K, indicating that the carbon-rich system can induce the high-$\hbox {T}_{{c}}$ value as well.

## Introduction

Carbon-rich material at high pressure and high temperature is interesting due to its outstanding properties to be super-hardness and superconductors. With regards to the study of boron carbides, a $\hbox {BC}_{1.6}$ phase has been observed using synchrotron-based X-ray diffraction, Raman spectroscopy, and energy-dispersive scanning electron microscopy^1^. The results have revealed that the *g*-$\hbox {BC}_{1.6}$ structure transforms to the diamond-like $\hbox {BC}_{1.6}$ at a temperature of 2230 K and a pressure of 45 GPa. They demonstrated that $\hbox {BC}_{{x}}$ can transform to hexagonal-$\hbox {BC}_{1.6}$ or orthorhombic-$\hbox {BC}_{1.6}$, depending upon the pressure and temperature conditions. However, the heating is not enough to make transformation to the cubic phase. Successfully, a cubic $\hbox {BC}_{{3}}$ structure has been synthesized at a pressure of 39 GPa and a temperature of 2200 K using a laser-heated diamond anvil cell,which the system is condensed by the $\hbox {sp}^{3}$ hybridization^2^.

In the progress for finding the word record of the highest superconducting transition temperature ($\hbox {T}_{{c}}$)^3^, the novel compositions in the interesting compounds have been reported at high pressure conditions. The hydrogen-rich materials of $\hbox {H}_{{3}}$S^4^ and $\hbox {LaH}_{{10}}$^5^ were proposed to be superconductor at gigapascal pressures (203 K and 250 K), which the high-$\hbox {T}_{{c}}$ temperature superconductivity depended on the conventional electron–phonon coupling and the shape of density of states around the Fermi level^6^. The high pressures above 100 GPa on these materials were required for structural stabilities of the rich hydrides. It was introduced that another material to find the better superconductors can be applied by the same concepts and methods^3^. Therefore, the high-pressure effect is one of the crucial tool for the formation of the atom-rich system, and the discovery of high-$\hbox {T}_{{c}}$ superconductivity. In another way to enhance the high-$\hbox {T}_{{c}}$ superconductivity, alkali-doped $\hbox {C}_{{60}}$ system has been presented as a superconducting material among carbon related compounds. The $\hbox {T}_{{c}}$ in the alkali-doped $\hbox {C}_{{60}}$ was reported as 33 K, which moderated by the coupling of electrons to high-frequency molecular vibrational modes^7^.

In the carbon-rich systems, the experimental study has been reported the synthesis of cubic $\hbox {BC}_{{5}}$ (c-$\hbox {BC}_{{5}}$), which is the diamond-like structure. The c-$\hbox {BC}_{{5}}$ was synthesized at a pressure of 24 GPa and a temperature of 2200 K using both laser-heated diamond anvil cell and large-volume multi anvil apparatus. The synthesized c-$\hbox {BC}_{{5}}$ showed that it is too high Vickers hardness ($\hbox {H}_{{v}}$) up to 71 GPa^8^. Moreover, the $\hbox {BC}_{{5}}$ phase is suggested as a candidate structure at high pressure through ab initio random search calculation. The $I{\bar{4}}m2$ structure was introduced as the most stable structure at high pressure. By the density functional theory (DFT) study, it was reported that the predicted $I{\bar{4}}m2$ structure is metallic, and the estimated $\hbox {T}_{{c}}$ of the superconducting phase is as 47 K^9^. In another class of boron carbides, the $\hbox {BC}_{{7}}$ was predicted as a candidate structure under high pressure using particle swarm optimization (PSO) methodology^10^. The $\hbox {BC}_{{7}}$ compound was the stable composition in the diamond-like $I\bar{4}m2$ structure, and also exhibited to be a super-hard material with the $\hbox {H}_{{v}}$ as 75.2 GPa. In addition, the effect of temperature has been presented as an influence for structural phase transformation of boron carbides such as $\hbox {BC}_{1.6}$ and $\hbox {BC}_{{3}}$, while the phase diagram boundaries of the boron carbides are still incomplete.

Keeping all these carbon-rich in mind, another class of carbides, the $\hbox {BC}_{{7}}$ phase predicted as a candidate structure under high pressure using particle swarm optimization (PSO) methodology^10^. Predictions of $\hbox {BC}_{{7}}$ revealed that the most stable structure is the diamond-like $P{\bar{4}}m2$ structure, and also exhibits superhard materials with the Vickers hardnesses of 75.2 GPa. They suggested that the diamond-like $P{\bar{4}}m2$ structure can be thermodynamically stable at ambient to high pressure (100 GPa). It is known that boron carbides are the most popular materials for experimental and theoretical investigations, in fact, the effect of temperature has an influence for structural phase transformation of boron carbides, such as $\hbox {BC}_{1.6}$ and $\hbox {BC}_{{3}}$.

Recently, the phase diagram of B-C system has been investigated from ambient to high pressure and under temperature effect^11^. The report showed that the effect of temperature is an important role for thermodynamically stable structure as well. In this work, the class of $\hbox {BC}_{{7}}$ phase is a point of interest for finding phase diagram with quasi-harmonics approximation (QHA), which can calculate thermodynamics properties also. Therefore, the main attention is to investigate the phase diagram of $\hbox {BC}_{{7}}$ and the structural searching analysis. The perspective of theoretical inspection displays a thermodynamically stable phase via the QHA calculation. The presence of route to high-Tc superconductivity via decomposition of binary diamond-like $\hbox {BC}_{{7}}$ compound is described through the Allen–Dynes equation.

## Computational details

The structure searching of the $\hbox {BC}_{{7}}$ was performed by the Universal Structure Predictor: Evolutionary Xtallography (USPEX) code^12^ combined with the Vienna ab initio simulation package (VASP) code^13^. In all subsequent generations, the random symmetric algorithm, which consisted of 40$\%$ heredity, 20$\%$ random symmetric, 20$\%$ softmutation, and 20$\%$ transmutation operators in the pressure range from 0 to 300 GPa with structures containing up to 4 formula units. All of the DFT calculations in this work used the generalized gradient approximation of the Perdew–Burke–Ernzerhof (GGA–PBE) functional^14^ for the exchange-correlation functional. We employed the projector augmented wave (PAW) method^15^, as implemented in the VASP code. The PAW potentials were applied with a plane wave basis set up to a cutoff energy of 700 eV and a $10 \times 10$ $\times$4 k-point mesh for the diamond-like $P{\bar{4}}m2$ structure, which was generated by the Monkhorst–Pack (MP) method^16^. The pseudocore radii of C and B are 1.1 Bohr and 1.1 Bohr, respectively, which are small enough that the overlap of spheres will not occur under applied pressure. All of the structural parameters were fully relaxed by using the Methfessel–Paxton smearing method^13^ and the conjugate gradient scheme. All considered structures were relaxed at each pressure until the Hellman–Feynman forces became less than 10$^{-3}$ eV/Å. The phonon calculation was calculated using the ab initio lattice dynamics with the linear response method as implemented in the VASP code together with the PHONOPY package^17^, which is an important role for investigation of the phase stability in metallic system^18–20^. The cutoff energy and k-point set for the phonon linear response calculation were used as 700 eV and $10 \times 10\times$4 for a $3 \times 3$ $\times$2 supercell (144 atoms) in the diamond-like $P{\bar{4}}m2$ structure at 250 GPa. We calculated elastic constants for the diamond-like $P{\bar{4}}m2$ structure, as implemented in the CAmbridge Serial Total Energy Package (CASTEP) code^21^. In the superconducting phase, we calculated the electron–phonon coupling (EPC) within the density functional perturbation theory^22^. via Quantum Espresso (QE) package^23^. The PAW potentials were employed in QE. The plane-wave energy cutoff of 60 Ry was used. The Brillouin zone (BZ) integrations in the electronic and phonon calculations were performed using the MP meshes. The EPC matrix elements were computed in the first BZ on $4 \times 4\times$2 q-meshes using individual EPC matrices obtained with a $24 \times 24\times$16 k-points mesh. The Allen–Dynes equation^24^ was used with the effective Coulomb pseudopotential parameter, $\mu$ $^{*}$= 0.10 (0.13) as follows;

1 $$\begin{aligned} T_{c} = \frac{\omega _{log}}{1.2} \exp \Big [ -\frac{1.04(1+\lambda )}{\lambda -\mu ^*(1+0.62\lambda )} \Big ], \end{aligned}$$

where $\omega _{log}$ is the logarithmic average of the spectral function, and $\lambda$ is the total electron–phonon coupling strength. We found that $\lambda$ value are less than 1.5 in most of our work, thus this form of Allen–Dynes equation is quite sufficient.

## Results and discussion

**Figure 1:**
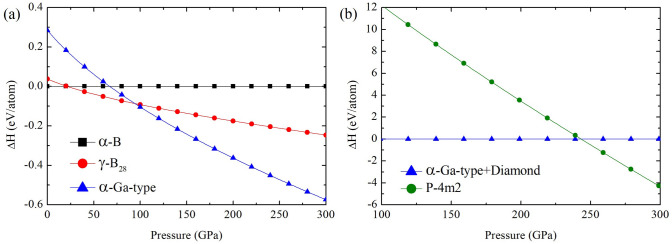
(**a**) The enthalpies-pressure relation of boron and (**b**) the enthalpies-pressure relation of $\hbox {BC}_{{7}}$.

**Figure 2:**
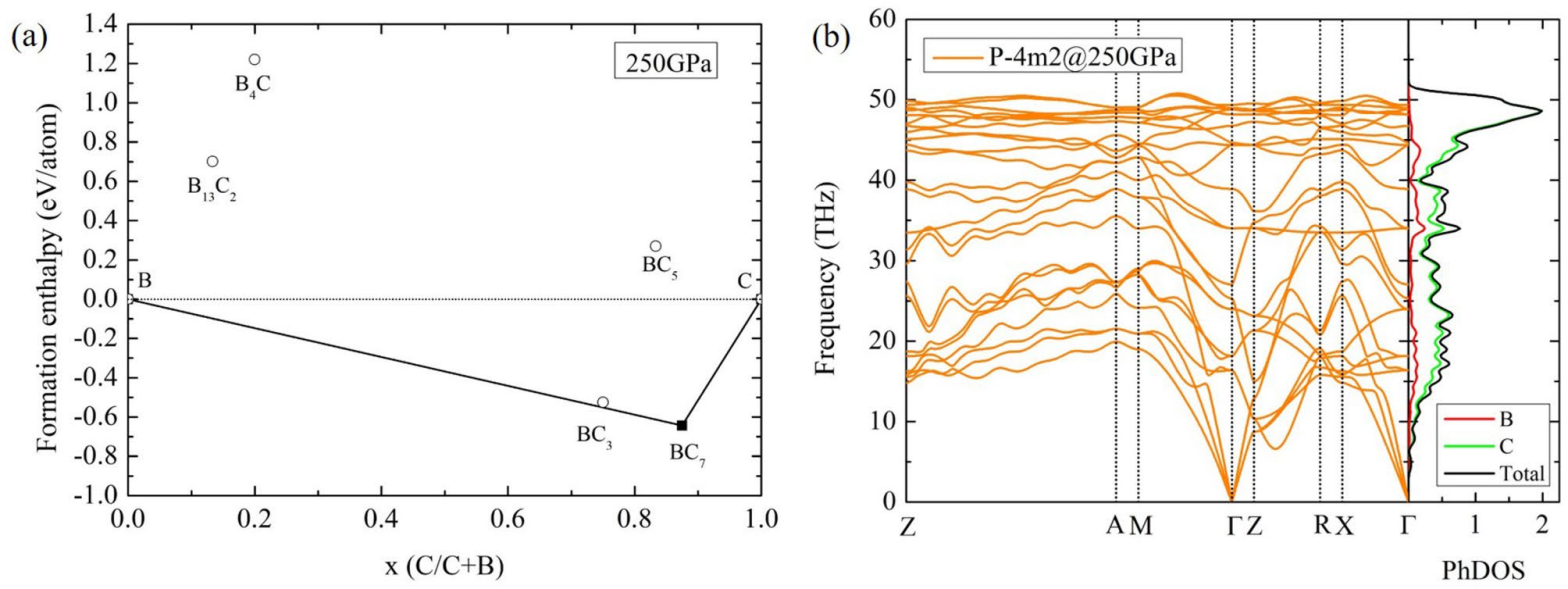
(**a**) Formation enthalpy of $\hbox {BC}_{{7}}$ presented in convex hull at 250 GPa and (**b**) the phonon dispersion and the phonon density of states of the diamond-like $P{\bar{4}}m2$ at 250 GPa.

To investigate the structural formation of binary diamond-like $\hbox {BC}_{{7}}$ compound, we present the thermodynamically stable structures of B and B-doped diamond in Fig. 1a,b. Structural phase transitions of B under high pressure are analyzed. The $\alpha$-B structure transforms to the $\gamma$-B structure at 26 GPa, and then it transforms to the $\alpha$-Ga-type structure at 98 GPa. The calculation displayed that the $\alpha$-Ga-type structure is the most stable structure (Fig. 1a). It is induced that the direct transformation from graphite to diamond structure at 15 GPa^25^. The diamond structure is the most stable structure and its stable structure can be used for identifying $\hbox {BC}_{{7}}$ structure. A predicted structure can be obtained from USPEX code. It is found that the decomposition of $\alpha$-Ga-type+diamond transforms to the diamond-like $P{\bar{4}}m2$. This is shown that the diamond-like $P{\bar{4}}m2$ structure can be formed above 244 GPa, as seen in Fig. 1b. The diamond-like $P{\bar{4}}m2$ structure is thermodynamically stable at pressure above 244 GPa, as shown in Fig. 1b. To investigate a formation of the diamond-like $P{\bar{4}}m2$ structure in BC7, it is focused on decomposition of B+C above 100 GPa. Convex hull of the B-C system is analyzed at 250 GPa which is the region of stability in the $P{\bar{4}}m2$ structure and the interesting pressure for $\hbox {T}_{{c}}$ calculation (Fig. 2a). The observable compositions of $\hbox {B}_{{13}}$ $\hbox {C}_{{2}}$, $\hbox {B}_{{4}}$C, $\hbox {BC}_{{3}}$, $\hbox {BC}_{{5}}$ and $\hbox {BC}_{{7}}$ are compared the formation enthalpy, which can determine the global minimum of structural stabilities in that pressure. The formation enthalpy ($\hbox {H}_{{f}}$) can be calculated as follows^26^:

2 $$\begin{aligned} H_{f} = \frac{H_{B_{x}C_{y}}-(XH_{B}+YH_{C})}{X+Y}, \end{aligned}$$

where $\hbox {H}_{{i}}$ represents the enthalpy of the $\hbox {i}th$ compounds in solid form, X and Y are positive numbers. By using the equation (2), the $\hbox {BC}_{{7}}$ composition presents the minimum of formation enthalpy in the B-C system at 250 GPa.

According to Fig. 2a, we also compute the phonon of the diamond-like $P{\bar{4}}m2$ structure at 250 GPa, which presents dynamically stable as well (Fig. 2b). All lattice vibration modes are positive. $\Gamma$-point is center of the Brillouin zone (BZ). The boundaries of BZ are given by planes related to points on the reciprocal lattice. The Z point is center of a face of the BZ, while the A point is edge of the BZ. Therefore, the path of ZA mainly represents the transverse acoustic (TA) and longitudinal acoustic (LA) phonons from the lattice vibration. The TA phonons (14–28 THz) are lower frequencies than the LA phonons (30–53 THz). These phonons correspond to shear sound waves for TA, and compressional sound waves for LA. In the diamond-like and zincblende structures^27,28^, it was suggested that the flattening of the TA phonon dispersion near the BZ edge can be explained with introducing long-range interatomic interactions, while the feature of TA and LA phonons in the ZA path exhibits nature of covalent bonds in this crystal. This effect is related to the oscillatory behavior in the phonon dispersion curves in cubic-3C silicon carbide system which gave high energy values of vibrational energies and chemical bonds, when compared with other systems^28^.

**Figure 3:**
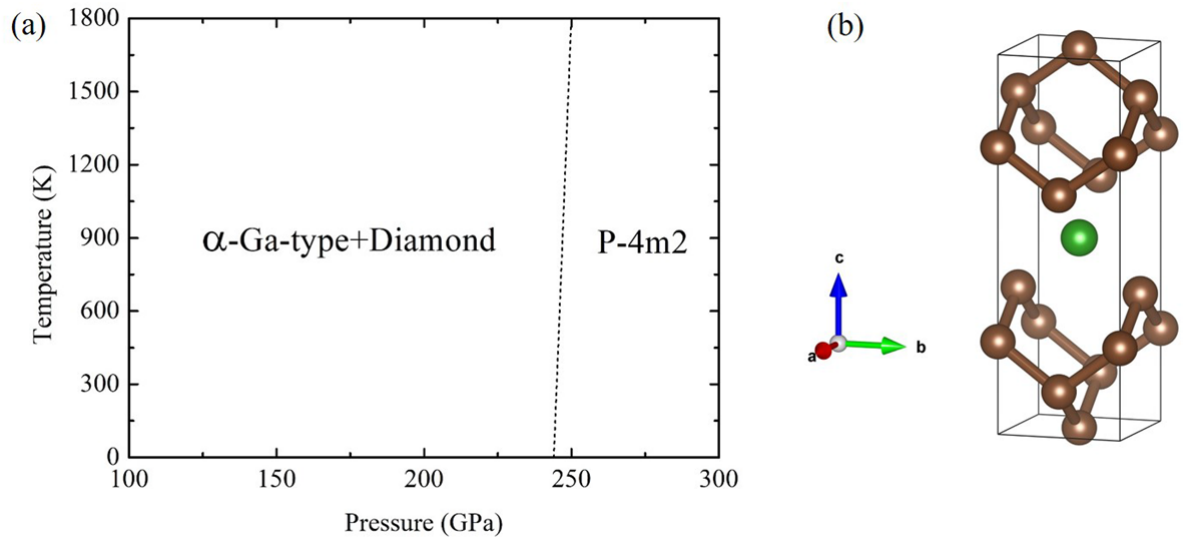
(**a**) Phase diagram of $\hbox {BC}_{{7}}$ and (**b**) crystal structure of the diamond-like $P{\bar{4}}m2$.

In addition, we construct a pressure–temperature (P–T) phase diagram to explore the diamond-like $P{\bar{4}}m2$ structure in $\hbox {BC}_{{7}}$ under temperature effect. The diamond-like $P{\bar{4}}m2$ is stable within the increasing of pressure and temperature monotonically (Fig. 3a). Figure 3b shows the crystal structure of the diamond-like $P{\bar{4}}m2$ structure.

**Figure 4:**
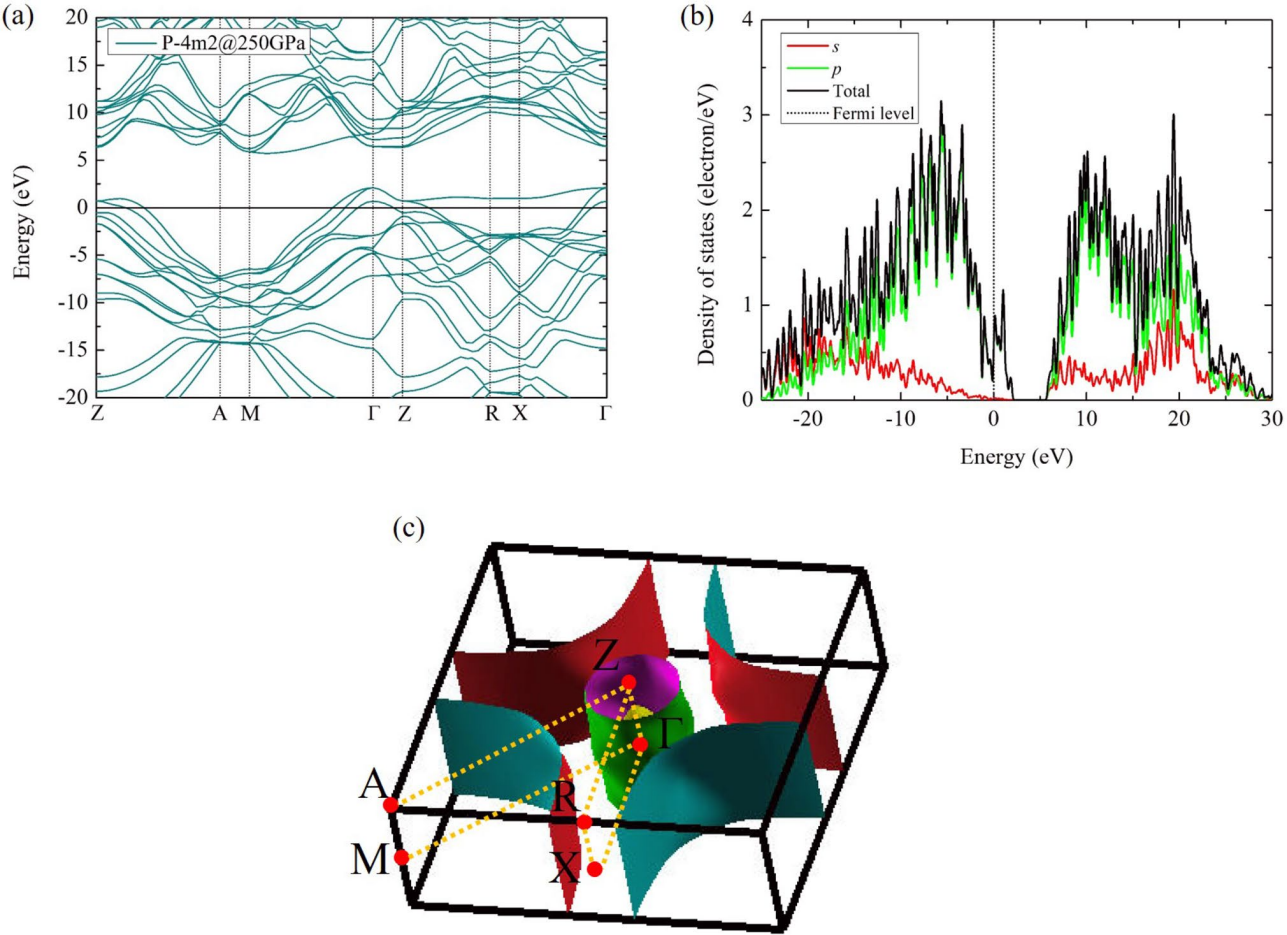
(**a**) Electronic band structure and (**b**) density of states of the diamond-like $P{\bar{4}}m2$ structure at 250 GPa and (**c**) Fermi surface of the diamond-like $P{\bar{4}}m2$ structure at 250 GPa.

The electronic band structure indicated the most significant result in this present study that the diamond-like $P{\bar{4}}m2$ structure is the metallic structure, in Fig. 4a. The remarkable solution displayed the flat band along the Z $\rightarrow$ R $\rightarrow$ X $\rightarrow$ $\Gamma$-point near Fermi level. Likewise, $\hbox {SH}_{{3}}$, $\hbox {YH}_{{10}}$, and $\hbox {LaH}_{{10}}$, these flat band exhibited high-$\hbox {T}_{{c}}$ superconductivity^29–32^. One of characteristic features to determine the high-$\hbox {T}_{{c}}$ compounds was consulted that it depends on the Van Hove singularities (VHS) around the Fermi level in the density of states^6^. The VHS around the Fermi level was introduced for characteristic feature of the high-Tc superconductivity in the $\hbox {H}_{{3}}$S compound^6^. They presented that the $\hbox {T}_{{c}}$ of $\hbox {H}_{{3}}$S is much higher than that of $\hbox {H}_{{2}}$S, which the VHS is absent in $\hbox {H}_{{2}}$S system.

In Fig. 4c, Fermi surface (FS) shows an occupied electron on a surface in reciprocal space, which was derived from the electronic band structure. We found that the FS displayed the large sheet along the Z $\rightarrow$ R $\rightarrow$ X $\rightarrow$ $\Gamma$-point (Fig. 4c), and corresponds to the band structure and density of state (Fig. 4a,b). From the electronic band structure and the FS viewpoint, it is worth noting that $\hbox {BC}_{{7}}$ is possible to discover a high-$T_{c}$ as well.

**Figure 5:**
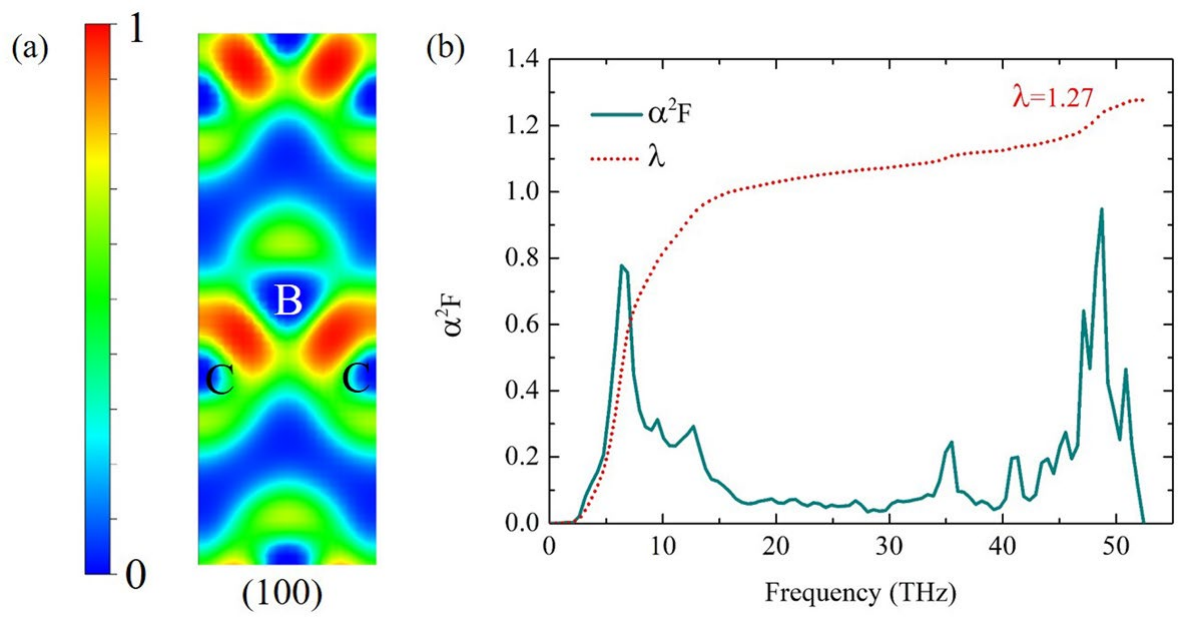
(**a**) The electron localization function (ELF) in the (100) atomic plane of the diamond-like $P{\bar{4}}m2$ structure and (**b**) the spectral function $\alpha ^{2}F$ (solid line) and the integrated $\lambda$ (dashed line) as a function of frequency of the diamond-like $P{\bar{4}}m2$ structure at 250 GPa.

We also compute a characteristic of bonding in the diamond-like $P{\bar{4}}m2$ structure using the electron localization function (ELF) method^33^. The ELF displayed the tendency of an accumulated electron^34–36^, with respect to a uniform electron gas of the same density. The (100) plane presents the contour plot of ELF and bonding at 250 GPa, which is a strong bonding due to an electron localization between first NN B–C is 1.449 Å(Fig. 5a). The remarkable results demonstrated metallic bond^9^ under the compression as well. At this point, it is worth nothing that the strong bonding may exhibit the high-$\hbox {T}_{{c}}$.

The chemical bonding and the existence of resonance structure of $\hbox {BC}_{{7}}$ can be described by the resonating valence-bond model^37^. The delocalization of states at valence band maximum relates to the multicenter bonding of the B-C bonds in $\hbox {BC}_{{7}}$. The B atoms in diamond-like structure are bonded to their neighbors through the hybridization of electronic states. The C-rich in the $\hbox {BC}_{{7}}$ system has a hole on the B sites, due to having the lower valence electrons of B. The superposition of the orbitals from B and C is expected to induce the electron–phonon coupling constant. The coupling electronic states could induce a resonating valence-bond superconducting state. The coupled frequencies of B and C atoms in $\hbox {BC}_{{5}}$ obtained from accumulation of electrons between B and C atoms^9^, relating to the systems of strong covalent bonding in $\hbox {H}_{{3}}$Se and $\hbox {H}_{{3}}$O^38^.

We also calculate bulk modulus (B) and shear modulus (G), which can be obtained from elastic constants ($\hbox {C}_{{ij}}$) by Voigt–Reuss–Hill (VRH) method^39,40^, as shown in the Table 1. The calculated $\hbox {C}_{{ij}}$ values show all positive, and satisfy with the Born stability criteria^41^, indicating the mechanical stability in the diamond-like $P{\bar{4}}m2$ structure at 250 GPa. The remarkable result of the $\hbox {C}_{{ij}}$ conforms the structural stability by phonon dispersion result. By calculated from the VRH method, it is obtained that B is 1157 GPa, and G is 649 GPa. In addition, Tian et al.^42^ suggested that the positive value of Vickers hardness ($\hbox {H}_{{v}}$) for an interesting material can be evaluated from B and G as follows:

3 $$\begin{aligned} H_{v} = 0.92 \Big (\frac{G}{B}\Big )^{1.137}G^{0.708}, \end{aligned}$$

By using this equation, the $\hbox {H}_{{v}}$ of $\hbox {BC}_{{7}}$ at 250 GPa is 46 GPa. The solution of $\hbox {H}_{{v}}$ indicates that the diamond-like $P{\bar{4}}m2$ presents the condition of super-hard material, which is related to the strong bonding between B and C.

**Table 1:** The calculated elastic constants ($\hbox {C}_{{ij}}$) of the diamond-like $P{\bar{4}}m2$ structure of $\hbox {BC}_{{7}}$ by Voigt–Reuss–Hill (VRH) method (in GPa).

$\mathbf{BC} _{{7}}$	P(GPa)	Elastic constants							B	G	$\hbox {H}_{{v}}$
		$\hbox {C}_{{11}}$	$\hbox {C}_{{12}}$	$\hbox {C}_{{13}}$	$\hbox {C}_{{16}}$	$\hbox {C}_{{33}}$	$\hbox {C}_{{44}}$	$\hbox {C}_{{66}}$			
$P{\bar{4}}m2$	250	2079	473	871	0	1839	900	388	1157	649	46

The calculated bulk modulus (B), shear modulus (G), and Vicker’s hardness ($\hbox {H}_{{v}}$).

**Table 2:** The calculated parameters and $T_{c}$ of $\hbox {BC}_{{7}}$, with solving the Allen–Dynes equations with $\mu ^{*}$= 0.10 (0.13).

Metal Carbides	Pressure (GPa)	$\lambda$	$\omega _{log}$ (K)	$T_{c}$ (K)
$^{a}$ $\mathbf{XeC} _{{2}}$	200	0.38	622	38
$^{b}$ $\mathbf{BC} _{{5}}$	0	0.89	810	47
$^{c}$ $\mathbf{NaC} _{{6}}$	0	2.92		127 (116)
$^{c}$ $\mathbf{AlC} _{{6}}$	0			$\sim$ 100
$^{d}$ $\mathbf{ClC} _{{6}}$	0			$\sim$ 40
$^{d}$ $\mathbf{NaC} _{{6}}$	25–75			> 100
$^{d}$ $\mathbf{AlC} _{{6}}$	25–175			> 100
$^{e}$ $\mathbf{BC} _{{7}}$	250	1.27	1498	164 (154)

^a^Refereence^36^.

^b^Refereence^9^.

^c^Refereence^43^.

^d^Refereence^44^.

^e^This work.

The spectral function $\alpha ^{2}F$($\omega$) of the diamond-like $P{\bar{4}}m2$ structure is shown in Fig. 5b, the $\alpha ^{2}F$($\omega$) referred the electron–phonon coupling (EPC) between an initial state $\hbox {k}_{{F}}$ and all other states on $\hbox {k}_{{F}}$. The calculated $\alpha ^{2}F$($\omega$) of B and C atoms contributed to the EPC. We investigated $T_{c}$ of the diamond-like $P{\bar{4}}m2$ structure using the Allen-Dynes modified McMillan equation^24^. We found that $\lambda$ is 1.27, $\omega _{log}$ is 1498 K, using $\mu$ $^{*}$= 0.10–0.13, and $T_{c}$ is 164–154 K at 250 GPa. The remarkable solutions manifested that the the diamond-like $P{\bar{4}}m2$ structure is the high-$\hbox {T}_{{c}}$. Likewise, binary carbon compounds with sodalite structure showed that $\hbox {NaC}_{{6}}$ is the high-$\hbox {T}_{{c}}$ among of them^43^. Addition, we explored the $\hbox {T}_{{c}}$ with the increased C atom, as seen in Table 2. We found that C-rich can enhance $\hbox {T}_{{c}}$ at high pressure; however, we suggested that the high-$\hbox {T}_{{c}}$ for carbon-rich materials may depend on the characteristic of bonding. Our results showed that carbon-rich materials is one of important effect for making the high-$\hbox {T}_{{c}}$ from the strong bonding between B and C-atom (Supplementary Information S1).

Our calculation results showed that the phonon stability of $\hbox {BC}_{{7}}$ can be observed $\sim$250GPa. In practical experiment, it is possibly to be found superconductivity of $\hbox {BC}_{{7}}$ at the lower pressure in the experiment, which was found in the shift-down of transition pressure in another material. This was discussed in the $\hbox {LaH}_{{10}}$ system^45^ that the quantum effects are important for the structural stabilities of solids with high electron–phonon coupling constants. The system could be destabilized by the large electron–phonon interaction, thus the reducing of critical pressure may be found in the synthesis.

## Conclusion

In this work, we investigate the decomposition of B–C, and employed the evolutionary algorithm for finding the stable structure at high pressures. The $\hbox {BC}_{{7}}$ in the diamond-like $P{\bar{4}}m2$ structure exhibits as the minimum of formation enthalpy in the convex hull, and it displays structural stability at pressure above 244 GPa, and under temperature effect. The strong bonding is observed from the electron localization function. The delocalization of states at valence band maximum corresponds to the multicenter bonding of the B–C bonds, which achieves the high temperature superconductivity in $\hbox {BC}_{{7}}$. In addition, the electronics properties, such as the band structure, density of states, and the Fermi surface, are discussed as the important key to reach the high-$\hbox {T}_{{c}}$ superconductivity for C-rich system at high pressure.

## Supplementary information

Supplementary Information
