# Supplementary material for: Route to high-Tc superconductivity of BC7 via strong bonding of boron–carbon compound at high pressure
Source: Sci Rep. 2020 Oct 22;10:18090. doi: 10.1038/s41598-020-75049-x (PMC7581753; doi:10.1038/s41598-020-75049-x)
Supplement: Supplementary file 1 — Supplementary Information [file 41598_2020_75049_MOESM1_ESM.docx]

**Supplemental Material**

**of**

**Route to high–T_c_ superconductivity of BC_7_ via strong bonding of boron–carbon compound at high pressure**

Prutthipong Tsuppayakorn-aek^1,2^, Xiaoyong Yang^3^, Prayoonsak Pluengphon^4^,

Wei Luo^3^, Rajeev Ahuja^3,5^, Thiti Bovornratanaraks^1,2^

^1^Extreme Conditions Physics Research Laboratory (ECPRL) and Physics of Energy Materials Research Unit, Department of Physics, Faculty of Science, Chulalongkorn University, Bangkok, 10330, Thailand

^2^Thailand Centre of Excellence in Physics, Ministry of Higher Education, Science, Research and Innovation, 328 Si Ayutthaya Road, Bangkok 10400, Thailand

^3^Condensed Matter Theory Group, Department of Physics and Materials Science, Uppsala University,

Box 530, SE-751 21, Uppsala, Sweden

^4^Division of Physical Science, Faculty of Science and Technology, Huachiew Chalermprakiet University, Samutprakarn 10540, Thailand

^5^Applied Materials Physics, Department of Materials and Engineering, Royal Institute of Technology (KTH), S-100 44 Stockholm, Sweden

**Computational details**

The phonon calculation was calculated using the ab initio lattice dynamics with the linear response method [1] as implemented in the VASP code [2] together with the PHONOPY [3] package which is an important role for investigation of the phase stability in metallic system. [4-6] The cutoff energy and k-point set for the phonon linear response calculation were used as 700 eV and 10x10x4 for a 3x3x2 supercell (144 atoms) in the diamond-like P-4m2 structure at 250 GPa.

**Result and discussion**

The solution of the linear response calculation manifested that it is similar to the supercell approach technique, as shown in Figure1S. The oscillatory behavior in the dispersion of the phonon bands displays all positive modes. Gamma-point is center of the Brillouin zone (BZ). The boundaries of BZ are given by planes related to points on the reciprocal lattice. The Z point is center of a face of the BZ, while the A point is edge of the BZ. Therefore, the path of ZA mainly represents the transverse acoustic (TA) and longitudinal acoustic (LA) phonons from the lattice vibration. The TA phonons (14-28THz) are lower frequencies than the LA phonons (30-53 THz). These phonons correspond to shear sound waves for the TA, and compressional sound waves for the LA. In the diamond-like and zincblende structures [7,8], the flattening of TA path near the BZ edge could be introduced by the long-range interatomic interactions, while the feature of TA and LA phonons in the ZA path exhibits nature of covalent bonds in this crystal. The calculated results from both linear response and super cell approaches presented clearly for the phase stability from lattice-vibrational modes, and the feature of oscillated frequencies due to the covalent bonding type and long-range interatomic interactions, which contained in the diamond-like P-4m2 phase.


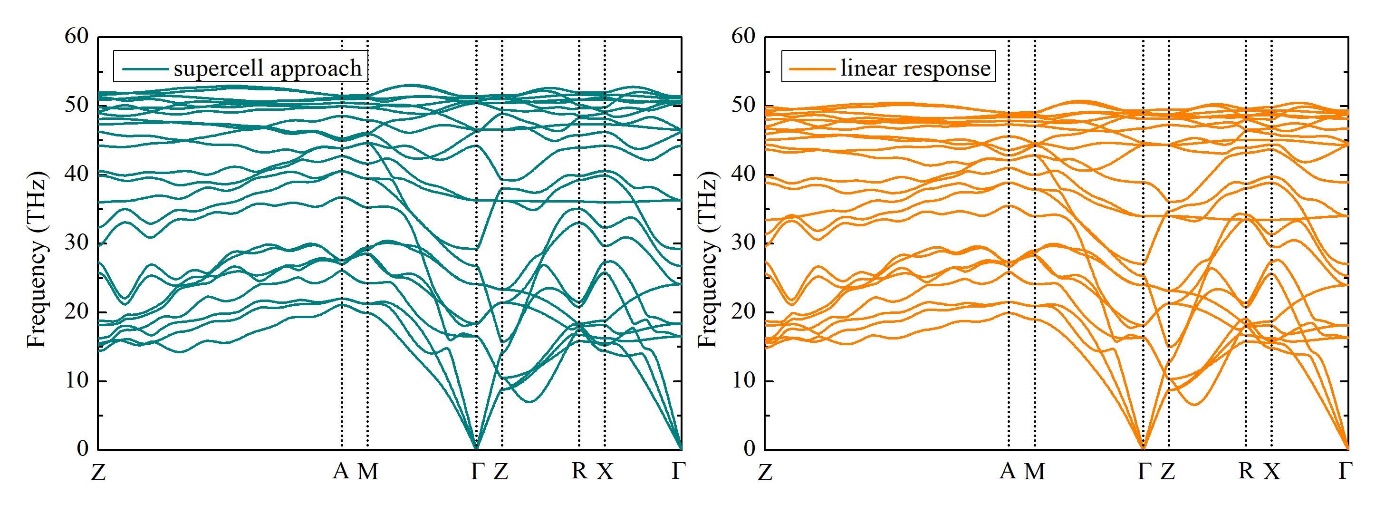


Figure 1S: The phonon dispersion of the diamond-like P4m2 at 250GPa.

**References**

[1] Baroni, S., de Gironcoli, S., Dal Corso, A. & Giannozzi, P. Phonons and related crystal properties from density-functional perturbation theory. Rev. Mod. Phys. 73, 515–562 (2001).

[2] Kresse, G. & Furthmüller, J. Efficient iterative schemes for ab initio total-energy calculations using a plane-wave basis set. Phys. Rev. B 54, 11169–11186 (1996).

[3] Togo, A. & Tanaka, I. First principles phonon calculations in materials science. Scr. Mater. 108, 1–5 (2015).

[4] Savrasov, S. Y. & Savrasov, D. Y. Electron-phonon interactions and related physical properties of metals from linear-response theory. Phys. Rev. B 54, 16487–16501 (1996).

[5] Ma, Y., Tse, J. S., Klug, D. D. & Ahuja, R. Electron-phonon coupling of α−Ga boron. Phys. Rev. B70, 214107 (2004).

[6] Haque, E., Hossain, M. A. & Stampfl, C. First-principles prediction of phonon-mediated superconductivity in xbc (x = mg, ca, sr, ba). Phys. Chem. Chem. Phys. 21, 8767–8773 (2019).

[7] Peter, Y. & Cardona, M. Fundamentals of semiconductors: physics and materials properties (Springer Science & Business Media, 2010).

[8] Varadachari, C. & Bhowmick, R. Ab initioderivation of a dataset of real temperature thermodynamic properties: Casestudy with SiC. Model. Simul. Mater. Sci. Eng.17, 075006 (2009).
